# Supplementary material for: Effect of a Single Multi-Vitamin and Mineral Supplement on Nutritional Intake in Korean Elderly: Korean National Health and Nutrition Examination Survey 2018–2020
Source: Nutrients. 2023 Mar 23;15(7):1561. doi: 10.3390/nu15071561 (PMC10097026; doi:10.3390/nu15071561)
Supplement: Supplementary file 1 [file nutrients-15-01561-s001.zip › nutrients-2281511-supplementary.pdf]

Table S1. Comparison of the percentage of intake inadequacy based on EAR between two groups

Nutrients	Male			Female		
	Below EAR			Below EAR		
	Food only	Food + MVMS	Change (%)	Food only	Food + MVMS	Change (%)
Vitamin A	80.3 (3.7)	58.6 (4.6)	-21.7	78.1 (3.9)	52.9 (4.7)	-25.2
Thiamine	21.0 (3.9)	0.4 (0.4)	-20.6	36.7 (4.9)	0.8 (0.8)	-35.9
Riboflavin	38.0 (4.6)	2.0 (1.2)	-36.0	44.2 (4.5)	2.2 (1.4)	-42
Niacin	46.1 (4.8)	17.5 (3.5)	-28.6	69.4 (3.8)	23.3 (3.9)	-46.1
Vitamin C	80.5 (3.5)	12.0 (3.0)	-68.5	82.4 (3.2)	6.9 (2.1)	-75.5
Calcium	67.3 (4.5)	54.2 (4.6)	-13.1	80.5 (3.5)	70.7 (4.5)	-9.8
Phosphorus	6.2 (2.0)	5.9 (2.0)	-0.3	32.5 (4.3)	29.2 (3.9)	-3.3
Iron	18.2 (3.8)	14.4 (3.4)	-3.8	21.2 (3.9)	11.4 (2.6)	-9.8

Abbreviation: EAR, estimated average requirement; MVMS, multi-vitamin and mineral supplements. Categorical variables wereexpressed as percentages and standard error (SE). Change (%) was calculated by subtracting the percentage of food only group from the 'food + MVMS' group.
